# Lessons Learned and Outcomes from Risk-Based Modernisation of Post-Mortem Inspection and Disposition Criteria of Beef, Sheep, Goat, and Pig Carcasses in Australia

**DOI:** 10.3390/foods13172775

**Published:** 2024-08-30

**Authors:** Andrew Pointon, Andreas Kiermeier, David Hamilton, Samantha Allan, Ian Jenson, Daryl Stevens, Ann McDonald, John Langbridge

**Affiliations:** 1 APFoodIntegrity Pty. Ltd., P.O. Box 7070, West Lakes, Adelaide, SA 5022, Australia; 2 Statistical Process Improvement Consulting and Training Pty. Ltd., P.O. Box 301, Gumeracha, SA 5233, Australia; andreas.kiermeier@gmail.com; 3 D Hamilton Consulting Pty. Ltd., 46 High Street, Willunga, SA 5172, Australia; david.hamilton@sa.gov.au; 4 Meat Export Branch, Department of Agriculture, Fisheries and Forests, Canberra, ACT 2601, Australia; samallan@iinet.com.au; 5 Meat & Livestock Australia, 1/40 Mount St., North Sydney, Sydney, NSW 2060, Australia; ijenson@firstmanagement.com.au; 6 Atura Pty. Ltd., Mount Martha, Melbourne, VIC 3934, Australia; daryl@atura.com.au; 7 Australian Meat Processor Corporation, Suite 2, Level 6, 99 Walker Street, North Sydney, Sydney, NSW 2060, Australia; a.mcdonald@ampc.com.au; 8 Teys Australia, Building 3, 2728 Logan Rd., Eight Mile Plains, Brisbane, QLD 4113, Australia; jlangbri@teysaust.com.au

**Keywords:** post-mortem inspection, disposition, risk, cattle, sheep, goats, pigs

## Abstract

The lessons learned from reviewing national risk assessments to modernise the Australian Standard for the post-mortem inspection and disposition judgement of beef, sheep, goat, and pig carcases are discussed. The initial risk profiles identified priorities for quantitative assessments. Broadly, the main difficulty encountered was the paucity of quantified performance for the current inspection. Resolving this involved acquiring gross abnormality data representing regional production/proportional abattoir volumes, the range of gross abnormalities appearing nationally, proportional occurrence at carcase sites, and seasonality to enable the comparison of procedures. The methodologies followed the Codex Alimentarius Commission’s risk assessment guidelines and are fully documented in the associated publications. The evidence and discussion are provided for the associated challenges experienced, including preventing contamination, the use of food chain information to support amendment, inspection as a part of industry Quality Assurance programmes, and opportunities to improve inspector training. The criteria considered by the Competent Authority for the determination of the equivalence of alternative post-mortem inspection techniques included comparisons of public health risk, non-detection rates for gross abnormalities, and microbial contamination resulting from inspection activities, as appropriate. Most of the gross abnormalities detected arose from animal health and welfare conditions affecting wholesomeness and did not present as food safety hazards. The non-detection rates between the current and alternative inspection (observation) were negligible. A quantitative risk assessment for *Cysticercus bovis* was conducted. Carcases with multiple gross abnormalities predominantly reflected historic infections (prior septicaemia), where trimming achieved wholesomeness unless they were cachexic.

## 1. Introduction

As a leading meat producer and exporter, the Australian industry maintains a proactive approach to assure food safety and product integrity from “paddock-to-plate” to protect public health and safeguard export market access [1]. Accordingly, a review of the organoleptic post-mortem inspection and carcase disposition criteria (PMID) in the *Australian Standard for Hygienic Production and Transportation of Meat and Meat Products for Human Consumption* (the standard) [2] capitalised on the opportunity and principles provided by the Codex Alimentarius Commission *Code of Hygienic Practice for Meat* [3].

The *Code of Practice for Hygienic Meat* [3] is a risk-based code, as is the Australian Standard [2], both responding to the World Trade Organization reforms in the mid-1990s providing for transparent regulations based on an assessment of risk and allowing alternative approaches that achieve the same outcome [4,5]. Furthermore, the Australian Standard, since the late 1990s, has provided the uniform, risk-based, outcome-oriented standard for the processing of domesticated meat species by all Australian (state and federal) Competent Authorities.

Australia’s beef industry in 2022 comprised 23.5 M extensively grazed cattle, producing approximately 1.9 million tonnes of carcase weight of beef and veal. From this herd, 2.7 million are grain-fed cattle being marketed (feedlot turnoff), equal to 47% of all adult cattle slaughtered. A total of 67% of beef and veal production was exported. Australia’s extensively grazed sheep meat industry in 2022 comprised 70 M animals producing 534,235 tonnes of lamb and 172,670 tonnes of carcase weight of mutton. In 2022, Australian lamb exports totalled 284,256 tonnes of shipped weight, and mutton exports totalled 144,004 tonnes. Australia’s goatmeat production in 2022 decreased by 7% from the previous year, totalling 14,795 tonnes of carcase weight from a total goat slaughter of 1,671,611 head of rangeland harvested animals [6]. As of June 2023, Australia’s pork industry was made up of approximately 285,000 sows, producing 5.6 million pigs per annum, resulting in 453,000 tonnes of pig meat each year with approximately 10% being exported [7].

While traditional organoleptic post-mortem inspection was developed in the late 19th and early 20th centuries to control important zoonotic diseases such as tuberculosis, anthrax, and taeniasis in Europe and North America, when these diseases were relatively prevalent [8,9], these inspection techniques have remained largely unamended in standards over the 20th century. However, the 21st century has seen substantial reform via the application of risk-based approaches to modernise post-mortem inspection and disposition judgement [10,11,12,13,14,15,16,17,18,19,20,21].

In addition to the international acceptance of risk-based reform, the opportunity for substantial modernisation in Australia has also been enabled by improvements in animal health and the reduced incidence and eradication of zoonoses over the past 40 years [22,23,24,25]. For example, Australia declared freedom from bovine tuberculosis in 1997 [26], and no cases of *C. bovis* were detected in a survey of 493,316 cattle in February 2008 [24]. Consequently, this review of the post-mortem inspection and disposition criteria (PMID) was initiated by industry, not the Competent Authority, as a response to any emerging or unmanaged meat safety risks in domestic or export markets attributable to Australian meat products.

This report provides an overview of conducting a risk-based review of the standard from both a technical perspective based on the types of evidence required for the determination of equivalence for a range of diseases and conditions, together with the lessons learned from applying the methodology at an industry level to modernise a national standard. It thus provides a review of the risk assessments and management considerations allowed by the Codex Alimentarius Commission [3] and an example of how the entire PMID system can be analysed by choosing appropriate methods and approaches. Full descriptions of the methodologies, data analysis, and interpretation that provide risk-based evidence for each amended procedure are presented in allied publications, which are cited accordingly.

## 2. Materials and Methods

### 2.1. Terms of Reference

The formal review of the Australian standard 4696:2007 [2] commenced in 2016.

The terms of reference (TOR) set by industry risk managers included the following:

- Removing techniques that are no longer necessary due to the improved animal health status of Australian herds and flocks;
- Altering or removing techniques where new knowledge of animal or foodborne disease indicates that current risk management techniques are not effective;
- Assessing the effect of contamination of edible tissues arising from current organoleptic post-mortem inspection (inspection) techniques;
- Reviewing disposition judgement criteria for total carcase condemnation where appropriate;
- Identifying techniques that are principally related to product quality rather than food safety that might be transferred to companies’ Quality Assurance systems.

### 2.2. Evidence-Based Approach

The scope of this review was restricted to organoleptic post-mortem inspection and carcase disposition criteria of the standard, which was not updated by a risk-based assessment when last reviewed in 2007 [2]. Carcase is defined as the entire body after bleeding that includes carcase parts, i.e., head and viscera (the carcase). In Australia, the Competent Authority determining the Australian Standard comprises state, territory, and federal meat safety jurisdictions. While the review intentionally utilised Australian data to amend the Australian standard, it applied and adapted methodologies reported internationally.

Taking a risk-based approach brought into play the following considerations:

- The need to quantify the performance of current techniques for red meat species in the first instance (i.e., establish current risk and/or non-detection rates of gross abnormalities affecting both food safety and wholesomeness) as a basis for comparison of alternative techniques;
- The increasing recognition that as traditional PMID techniques are insufficient to prevent and control the microbiological risks of illness associated with consumption of meat, and the potential for counter-productive microbiological contamination of edible tissues resulting from the actual PMID techniques is attracting regulatory attention [10,11,12,27,28];
- Increased use of information “up and down” the supply chain to inform both disease control and PMID [20];
- Recognition that PMID has a dual function, serving animal health and public health objectives that need to be preserved [3,14].

**Figure 1:**
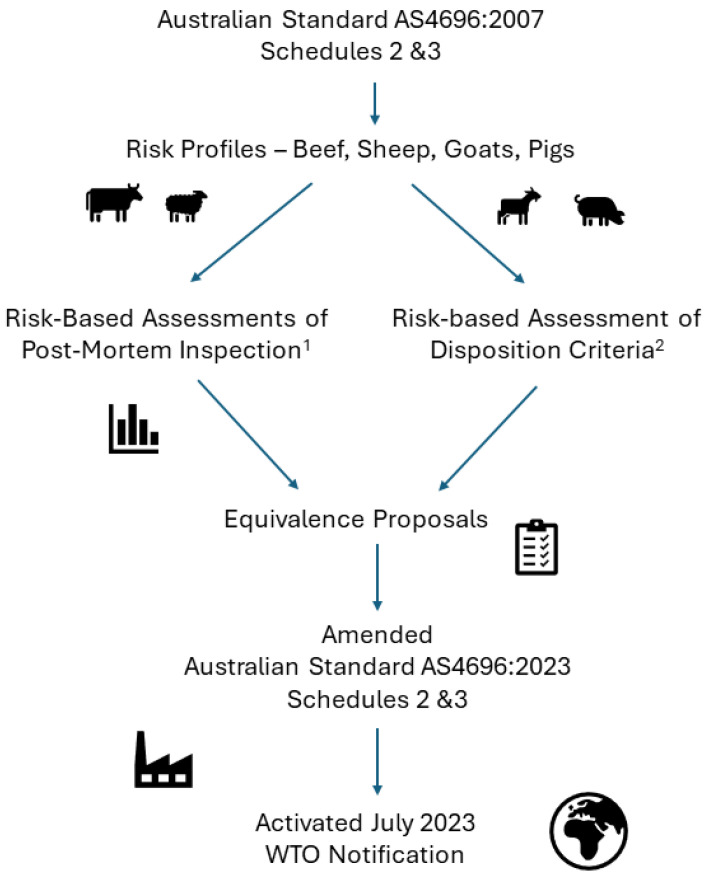
Risk-based process to amend the Australian Standard for post-mortem inspection and disposition judgement of beef, sheep, goats, and pigs. ^1^ [27,29,30,31,32,33,34,35,36]; ^2^ [37,38,39,40].

The review commenced with two risk profiles for cattle/sheep/goats and pigs (Figure 1) to identify hazards and rate the risk associated with hazard–gross abnormality combinations [25]. These reflected assessments reported for the United Kingdom [15,16]. The first step involved examination of foodborne illness databases to identify contemporary meat-borne hazards. The process then assembled data from research reports on the occurrence and prevalence of identified hazards across the meat supply chain into an exposure assessment framework [3]. Fourteen specific priorities were identified; seven were used to quantify and compare the effectiveness of alternative inspection techniques with the standard, and seven assessments of criteria were used for carcase disposition judgements (Figure 1).

To compare the effectiveness of alternative inspection techniques, non-detection rates of gross abnormalities were extensively used as an indicator of consumer risk, i.e., as a surrogate Acceptable Level of Protection (ALOP). This quantitative comparison was applied to gross abnormalities that only affected wholesomeness and those affecting food safety [25,29,30]. For *Cysticercus bovis*, a full quantitative risk assessment was conducted to estimate the effect of amended inspection on consumer risk [31]. Comparisons of microbial contamination rates between inspection techniques were presented as evidence for amendments proposed for acceptance of equivalence [27,29].

### 2.3. Approaches to Compare the Performance of Alternative with Existing Post-Mortem Inspection Techniques

To compare the effectiveness of detection of gross abnormalities by alternative techniques, knowledge of the *Se* of detection of gross abnormalities by inspection was an important starting point. However, it became immediately apparent that there was a paucity of sensitivity (*Se*) data for current inspection techniques for cattle, sheep, and goats determined by studies undertaken in Australian abattoirs. Consequently, quantification of the performance of current inspection techniques was included in project methodology to enable comparison with alternative techniques.

This Sensitivity (*Se*) data gap, however, did not apply to inspection of pigs where the assessment of equivalence of routine visual-only inspection utilised field data from Denmark and Australia [29,32,41]. These *Se* data were used in conjunction with more recent data on prevalence of gross abnormalities in Australia to compare the non-detection rates of gross abnormalities associated with routine visual-only inspection with the standard.

In this Australian review, several approaches to address these *Se* data gaps were employed. These included the following:

- Quantitative risk assessment for C. *bovis* where unconfirmed gross abnormalities were conservatively modelled as positive for *C. bovis* using published *Se* data [31];
- Additional veterinary specialist inspection to provide a gold standard prevalence of gross abnormalities, i.e., including those not detected by current and alternative inspection [29,42];
- Use of Delphi expert opinion [13,30];
- Only comparing non-detection rates when the initial prevalence of gross abnormalities affecting food safety was estimated as negligible, i.e., the vast majority detectable abnormalities only affect wholesomeness [25,33,37,43].

To enable comparison of the non-detection rates, the prevalence of grossly detectable abnormalities at slaughter had to be determined in most assessments. The sampling framework for prevalence surveys applied exposure assessment principles, representing the spectrum of production systems, breeds, and regional production and processing numbers across Australia on a proportional basis and including seasonality and animal age when relevant [25,30,34,35].

To acquire nationally representative data to meet the requirements of a national standard, data recording was undertaken by specially trained Australian Government Authorised Officers. Data acquisition and recording methods are detailed in each of the published reports. The projects comparing inspection techniques for pigs were primarily implemented and managed in abattoirs by veterinarians that are authors of this report. For the historic major zoonoses of cattle, tuberculosis and *C. bovis,* suspect samples were submitted for laboratory testing [23,24].

### 2.4. Contamination and Net Effect of Post-Mortem Inspection

Application of the CAC risk assessment guidelines [3] bring into scope an examination of the likely negative effect of traditional inspection where the net effect may be a poorer food safety outcome. A negative net effect is where the detection and removal of foodborne hazard–gross abnormality is outweighed by contamination of edible tissue with hazards resulting from the actual inspection techniques utilised [11]. Australian data were used to model net effects [32,38].

Additionally, a prevalence survey in two pig abattoirs was undertaken to determine the contamination rate of inspectors’ hands by *Salmonella* spp. during routine carcase inspection [25].

### 2.5. Techniques Transferable to Companies’ Quality Assurance Systems

One of the TOR was to identify procedures that were principally related to product quality rather than food safety, which might be transferred to companies’ Quality Assurance systems.

To examine this proposition, a study was conducted to establish the *Se* of detection of pleurisy in pigs by routine slaughter-line personnel when removing thoracic organs [44]. Pleurisy of pigs was chosen for this assessment as it is not associated with foodborne hazards and is a leading reason for partial condemnation of pigs in Australia [45]. This leads to processing inefficiency due to excessive numbers of carcases detained for trimming.

An assessment compared detection of pleurisy on the same carcases by experienced slaughter-line personnel at routine evisceration with official PMID.

### 2.6. Rationale for Assessment of Criteria Used for Carcase Disposition Judgement

During the conduct of the initial risk profiles the project team was advised that carcase condemnation rates for some species at some abattoirs were substantially greater than the national rates. These scenarios enabled opportunistic case studies of affected carcases at these abattoirs to evaluate the effectiveness of carcase disposition criteria (Figure 1), which included the following:

- Discernment of acute from chronic carcase abnormalities;
- Disposition outcomes for carcases with multiple, chronic abnormalities (prior septicaemia);
- Interpretation of terms such as “*systemic effects*” to describe the extent of the disease or condition [2].

For many gross abnormalities that result from systemic infections, the aim of inspection is to determine the actual stage of infection the carcase lesions represent in the continuum from acute (current systemic), organising (infection localising to affected sites), resolving (localising lesions), to chronic (lesions localised and then diminishing in extent until no residual tissue damage remains), with or without sequelae (Table 1). Each subsequent stage is associated with a decreasing probability of the causative organism still being present. The duration of each stage will vary considerably from animal to animal and will be further influenced by the effectiveness of treatments.

Knowledge of gross abnormalities associated with foodborne hazards is also paramount in determining PMID techniques. Consequently, the opportunity to provide information to assist inspectors was undertaken with the aim of reducing uncertainty in determining carcase disposition; this was a need heightened by the short time-frame available for routine inspection.

Accordingly, microbiological examination of gross abnormalities, lymph nodes and edible tissues were undertaken to provide additional hazard-based information to supplement pathology criteria in determining carcase disposition [32,37,39,40], replicating those developed for pig carcases affected by embolic pneumonia in Denmark [17]. The approach not only considered detection of the infectious agents that may be causing the primary abnormalities and septicaemia (i.e., in non-draining lymph nodes), but also whether foodborne hazards (e.g., *Salmonella* spp.) occur in gross abnormalities and edible tissues as a sequel to the primary illness.

Priorities for the assessment of disposition criteria included pneumonia/pleurisy complex and polyarthritis of cattle and pig carcases. Cattle and pig carcases fitting the case definition, leading to total condemnation due to multiple gross abnormalities and not showing signs of cachexia, were subjected to the following assessment and testing regime:

- Description of gross abnormalities and carcase sites affected;
- Assessment of the acute versus chronic nature of gross abnormalities;
- Microbiological testing of the following:
  ○ The primary lesion for causative infectious organisms;
  ○ Lymph nodes not directly draining the lesion/abnormality and edible tissue to determine if totally condemned carcases were septicaemic (i.e., acute systemic infection);
  ○ To assess of the presence or absence of food safety hazards in edible tissue.

### 2.7. Expert Panels

Expert panels with extensive experience provided practical guidance on survey methodologies to estimate prevalence to fill data gaps assisted in interpretation of key findings and provided information on practical design of validation projects conducted during routine slaughter operations [25,30]. The capabilities of the expert panels were as follows:

- Experience with regulatory reform using Codex Risk Assessment guidelines;
- Practical and long-standing field experience with meat inspection at the operational and plant management level;
- Experience in Competent Authority roles (including domestic standards management and market access considerations);
- Veterinary experience in the field as an abattoir veterinarian and respected authorities in their field;
- Experience in using published risk rating methods and publishing outcomes of related studies;
- Awareness of the level of evidence required by Competent Authorities to assess equivalence;
- Statistical and epidemiological skills to underpin data rigour.

## 3. Results and Discussion

### 3.1. Hazard Identification

The Hazard Identification (HI) demonstrated *Salmonella* Typhimurium as the most likely hazard to occur in association with fresh meat products in Australia for these species. However, *Salmonella* spp. was found to be a primary agent or secondary contaminant of a minority of gross abnormalities. Conversely, this Australian HI confirmed that most gross abnormalities found at slaughter, in both type and prevalence, are not associated with identified foodborne hazards and only affect wholesomeness that result from animal health and welfare issues [25].

*Staphylococcus aureus* was identified as being a common cause of meat-borne illness in Australia with outbreaks attributed to human strains contaminating post-cooked product with subsequent temperature abuse enabling toxin build-up [25], as reported by EFSA [10,11,12]. However, *Staphylococcus aureus* is commonly associated with gross abnormalities of carcases affecting wholesomeness and were considered accordingly in the review. A similar rationale was also found to apply to *Clostridium perfringens*.

### 3.2. Findings and Priorities from Risk Profiles

The initial risk profiles conducted for the review found few reports on the *Se* of the post-mortem inspection conducted in Australia [25], though some were available from EU reports [10,11,12,31,41]. Similarly, there is a paucity of prevalence surveys based on regional production/slaughter volumes and the range and prevalence of gross abnormalities occurring at specific carcase sites (e.g., liver, lymph nodes, etc.) needed for a comparison of alternative inspection techniques. A review of the published data indicated that most gross abnormalities detectable in beef, sheep, goat, and pig carcases in Australia only affect wholesomeness [25].

Conducting the risk profiles identified fourteen priorities for a risk-based assessment of PMID activities for cattle, sheep, goats, and pigs that addressed the questions posed by risk managers, including the following:

- Removing post-mortem inspection procedures that are no longer necessary due to the improving animal health status of Australian animals, e.g., bovine tuberculosis, *Cysticercus bovis* and caseous lymphadenitis of sheep and goats;
- Altering or removing procedures where new knowledge of animal or foodborne disease indicates current risk management procedures are not effective, e.g., inspection of spleens and unenucleated kidneys of sheep and goats;
- Assessing the effect of inspection on microbial contamination of edible product, e.g., visual inspection of offal of sheep, goats, and pigs;
- Reviewing the criteria used to determine the disposition e.g., melanoma of pigs, pneumonia/pleurisy of cattle and pigs, and polyarthritis of cattle and pigs;
- Identifying procedures that are principally related to detecting gross abnormalities that affect product wholesomeness rather than food safety and might therefore, be managed within Quality Assurance arrangements, e.g., pleurisy of pigs.

### 3.3. Equivalent Alternative Post-Mortem Inspection Techniques

The criteria for assessing the equivalence of alternative inspection is set by the Competent Authority (Australian Meat Regulators Group). These include assessment of any adverse effects on food safety, wholesomeness, animal health (including zoonoses), and animal welfare. The key quantitative metrics applied for the determination of equivalence of inspection techniques included comparisons of estimated public health risk, non-detection rates, and microbial contamination, as appropriate.

In summary, the validation studies of alternative techniques based on national data demonstrated the following negligible effects:

- Differences in non-detection rates between observation and palpation, i.e., undetected abnormality increase commonly in the order of 1/1000 to 1/10,000 carcases, i.e., predicted negligible difference in effectiveness after amendment;
- Occurrence of gross abnormalities of foodborne significance;
- Adverse effect on information available for carcase disposition judgement resulting from visual-only inspection;
- Adverse effect on animal health and welfare surveillance.

The amended equivalent inspection techniques in AS4696:2023 Schedule 2 [46] from applying an equivalence assessment include the following:

- Reduced incision of lymph nodes for bovine tuberculosis [23,47];
- Cessation of routine incision of masseters for *Cysticercus bovis* of cattle unless flagged as high-risk, wherein full carcase inspection is required. Otherwise, routine incision of hearts is retained [24,31,46,48,49];
- Reduced palpation of lymph nodes for detection of caseous lymphadenitis of sheep and goats [27,30];
- Visual inspection of sheep and goat spleens instead of palpation, irrespective of being kept for human consumption [36];
- Routine visual-only inspection of commercially reared pigs including cull breeding stock, irrespective of whether they are reared in intensive and extensive production systems [29,32,34];
- Observing unenucleated kidneys of sheep, goat, and pig kidneys when not for human consumption and observation of enucleated kidneys when for human consumption [33,35].

### 3.4. Retention of Palpation and Incision

While routine incision and palpation is significantly reduced for cattle, sheep, and goats and is removed for pigs, a provision is added for the use of palpation and incision “where appropriate” for all detected or suspected conditions (Table 4, Schedule 2 AS4696:2023; [46]), as determined by the European Commission [50]. For example, in moving to the routine visual-only inspection of pigs, such circumstances would involve suspect carcases identified through the visual detection of relevant abnormalities or herd health history particularly evidenced by recent PMID data [51]. This approach is especially relevant to modern pig production in Australia, whereby sale pigs are shipped directly to the same abattoir from the same farm a weekly basis. It also applies to lot-fed cattle where site related conditions may emerge, with carcases showing similar gross abnormalities at the same abattoir [39,40]. The provision of “where appropriate” in the amended standard allows varying PMID techniques in real-time.

In keeping with the risk-based approach, when palpation and incision are used, these additional techniques must be followed by an effective decontamination of hands and associated equipment to minimise the subsequent contamination of edible tissue [46]. These assessments of equivalence and amended procedures mirror the risk-based modernisation of PMID occurring internationally in recent years [10,11,12,18,19,50].

However, routine palpation and/or incision is retained in some circumstances for cattle, sheep, and goats. Routine palpation is retained for the lungs, liver, kidney, and tongue to detect deep-seated gross abnormalities, e.g., abscesses, granulomas, etc. Palpation and incision also remain unchanged if specified diseases are detected or suspected, notably tuberculosis and parasitic zoonoses (Table 4, Schedule 2 of AS4696:2023; [46]). For cattle and buffalo, the incision of masseters is only required under specified circumstances [46,48]. For sheep and goats, while palpation is significantly reduced due to reduced palpation for caseous lymphadenitis, the hearts are routinely palpated and bile ducts are incised (Supplementary Material 1, Tables A7.1–A7.4; [25]).

### 3.5. Use of Food Chain Information

In terms of food chain information (FCI) being available from primary production to tailor meat hygiene requirements [3,20,21], three forms of this are practised or are developing in Australia. The first applies to all consignments of cattle, sheep, goats, and pigs slaughtered for human consumption in Australia. These are accompanied by a mandated statutory vendor declaration of relevant food safety and traceability information [52], which cover suspect bovines for *C. bovis*, animals known to be carrying physical hazards (e.g., broken needles), and any that do not meet the chemical residue withholding periods [53,54].

The second example of routinely practised FCI is where pre-slaughter information is used to guide PMID inspection for *C. bovis* [46], a principle advocated by Jacobs et al. [21]. Low-risk cattle now require the routine inspection of observation of masseters and routine incision of heart muscle for *C. bovis*, noting that while hearts are the most common site for CB, the retention of incision is primarily for the detection of hydatids. For high-risk groups of cattle, defined as the animal has grazed properties where exposure to *Taenia saginata* eggs may have occurred, full carcase inspection applies. This requires the incision of masseter muscles, heart, and other sites (AS4696:2023, Schedule 2, Table 4: [46]).

An accompanying revised CB risk management framework of FCI provides the detail required by regulators, processors, producers, and other stakeholders along the supply chain about the actions and requirements to ensure risk-based PMID is applied to cattle classified as low- or high-risk for *C. bovis* [48,49].

This amended PMID for CB also applies to cattle exposed to adequately recycled human sewage water, ensuring that the risk is identified and managed appropriately. Cattle that grazed on lands treated with recycled water that has been treated with a helminth egg Log Reduction Value (LRV) of ≥4.0 or LRV 3.0, if an alternate clean water source is available for cattle to drink (i.e., no access to recycled water for livestock to drink), are now managed as low-risk cattle for PMID [48,49,55,56,57].

The third form of FCI being implemented in parallel to the PMID review is the eventual adoption of the reporting of information of gross abnormalities encountered at routine slaughter back to producers for pigs [45,51] and sheep and goats [58]. This approach is especially suited for the extensive rangeland production systems practised in Australia for cattle, sheep and goats, while reflecting the intent of the Codex-facilitated reform (3).

### 3.6. Contamination and Net Effect of Post-Mortem Inspection

In the 2011 EFSA review of the post-mortem inspection of pigs [11], it is noted that hygiene, which is a pre-requisite for the production of safe meat, is also hampered by manual meat inspection procedures. Making further incisions in tissues/organs following the incision of even “normal appearance” lymph nodes is a pathway for spreading pathogens such as *Salmonella* spp. and *Yersinia enterocolitica* over the carcass and between carcasses [32,59].

In the view that the bulk of the observable abnormalities in Australian slaughtered stock are not of food safety significance [25], an assessment was undertaken to model whether inspection may be microbiologically counter-productive. Using previous Australian data to model the net effect, Pointon et al. [32], (Appendix 2 [38]) estimated that the visual-only inspection of lymph nodes of pigs’ heads outweighs the incision by around 214 to 1, i.e., for each *Salmonella*-contaminated head lymph node abscess detected, 214 carcases (subsequent sites) handled are potentially contaminated. The ultimate effect on consumer risk was not estimated. Since the publication of the previous standard [2], there has been a provision to observe, excise, or discard these lymph nodes, and that provision has been retained [46].

To further examine the potential contamination pathways, a prevalence survey in two large pig abattoirs found that 14% of inspectors’ hands were contaminated by *Salmonella* spp. during routine carcase inspection under the previous standard [25]. This is consistent with evidence of faecal contamination of these inspectors’ hands in the survey, with *E. coli* at 50% of samplings and having a mean log count of 2.23 cfu/swab. By comparison, 9.3% of meat handlers tested positive for *Salmonella* spp. in Portuguese abattoirs [60].

However, inspection is only one of many processes involved in carcase processing that may contaminate edible tissues. While reports indicate the increase in bacterial counts due to inspection is generally low, increases in the prevalence of hygiene indicator organisms and foodborne hazards from inspection may be substantial [27,28].

### 3.7. Techniques Transferable to Companies’ Quality Assurance Systems

The assessment of the ability of experienced slaughter-line personnel to detect pleuritic carcases in pigs at routine evisceration found a high detection rate (*Se* 0.9). This supports the implementation of preliminary carcase trimming as part of routine carcase dressing (i.e., partial stripping of affected pleura before official PMID) to achieve carcase wholesomeness. This approach minimises carcases having to be detained for the final trimming. In addition, there is no loss of information to inform carcase disposition as partially stripped pleura (i.e., dangling in situ), which are obvious to official inspectors when subsequently making the final carcase disposition judgement [44].

These findings offer feasible improvement in efficiency for both processing and official carcase inspection and have been incorporated in abattoir Approved Arrangements without changes to the standard.

### 3.8. Amended Carcase Disposition Judgement Criteria

During the review, the awareness of relatively high total carcase condemnation rates for beef became apparent at some abattoirs. This provided the opportunity for intensive carcase evaluations at these abattoirs to investigate the reason for high rates of carcase condemnation. Similar investigations were conducted for pig carcases, while desk-top studies were employed for sheep and goats as official meat safety risk managers required data for each species.

The consistent finding was that carcases with multiple gross abnormalities mostly reflected historic infections presenting as chronic phase lesions that are no more than a historical infection (i.e., prior septicaemia), which could be trimmed to achieve wholesomeness of meat for human consumption as proposed by Murray in 1986 [61] and reported in beef by Petersen et al. in 2022 [62].

These assessments of the disposition criteria, which included pneumonia/pleurisy complex and polyarthritis of cattle and pig carcases, found that those with multiple chronic lesions mostly harboured primary infectious agents in the primary site, but not peripheral lymph nodes or edible tissues. *Salmonella* spp. was not recovered from any of these sites in the beef and pig carcases investigated [37,39,40].

Consequently, to minimise the uncertainty in judging the final carcase disposition, the use of acute or chronic classification is now consistently applied across these gross abnormalities in the standard. Amended equivalent disposition criteria now published as AS4696:2023 Schedule 2 [46] for arthritis and pneumonia/pleurisy now includes the following:

- Replacement of “*Systemic effects*” [2] with specific descriptions and disposition for acute (disease manifest as septicaemia, indicated by petechial haemorrhages and/or polyserositis) or chronic (may show multiple localise abnormalities of lungs/joints, no signs of septicaemia) abnormalities;
- Multiple chronic abnormalities not being interpreted as “*Systemic effect”* [2] and thereby a reason for total carcase condemnation;
- These carcases may now be trimmed to achieve wholesomeness instead of being totally condemned when not showing generalised signs of septicaemia and/or cachexia.

Where uncertainty remains, particularly for high value beef carcases, additional testing to determine safety and wholesomeness may be undertaken to minimise unnecessary wastage [46].

It is noted that the total carcase condemnation rates varied unexpectedly across abattoirs processing comparable cohorts of beef carcases (e.g., lot-fed cattle). The results suggest that, at some abattoirs, the judging of the final carcase disposition may have taken on a site interpretation and inspection culture that could benefit from objective data from carcase investigations [39,40], as noted by Petersen et al. [62] in Denmark.

Training for meat inspectors needs to include, for example, the continuum of clinical signs, gross abnormalities, and microbiology of diseases and conditions (e.g., bovine respiratory disease) from acute infection to the localisation of the pathology leading towards resolution, with or without sequelae (Table 1, [39]). Similar results were obtained for the intensive testing of beef and pig carcases with arthritis [37,40]. It is noted that the carcases investigated in these studies were predominantly at the resolving (localising) and chronic stage, even though the case definition for the carcase selection was total carcase condemnation determined at routine inspection. The information provided is intended as a guideline to better define and minimise the number of carcases where uncertainty remains for the final disposition judgement [39].

For melanoma in pigs, 1.53% of the 130,000 pigs surveyed had visible melanoma lesions on the skin. Of these 1989 carcases with skin melanoma, 2.86% had related lesions in the immediate draining lymph nodes. None showed progression beyond these immediate lymph nodes ([38]; Appendix 4). For the specific condition of melanoma of pigs, the amended disposition is “depending on extent, lesion trimmed and condemned or affected carcase part condemned” [46]. This is now consistent with disposition judgements in other countries for this inherited gross abnormality predisposition.

## 4. Conclusions

By acquiring risk-based national data, the review has validated that the bulk of gross abnormalities detected by post-mortem inspection in Australia affect wholesomeness, not food safety. This is largely attributed to improved animal health and the elimination of meat-borne zoonoses over the past 40 years. Consequently, it is unsurprising that the review was initiated by industry, not as a jurisdictional response to any emerging or unmanaged food safety risk. These findings for Australian production systems substantiate other evidence that post-mortem meat inspection serves to detect gross abnormalities that are almost entirely related to food quality and on-farm (health and welfare) management issues [10,11,12,13,14,15,16,18,21,63]. Consequently, a substantial number of amendments to the PMID were long due, notably for *C. bovis* [31,48] and caseous lymphadenitis of sheep and goats [30].

There were many lessons learned from performing this risk-based review. For the most part, a quantitative assessment of the performance of the current PMID found frequent data gaps, which had to be quantified to compare the performance of an alternative PMID. To address this fundamental gap, projects established the prevalence of gross abnormalities across large data sets that were based on regional production and proportional abattoir volumes, the range of gross abnormalities occurring across the continent, and age and seasonality where relevant. These also determined the proportional occurrence of gross abnormalities on an organ/carcase site as baseline data for determining the effectiveness of the detection by post-mortem inspection. These data sets reported gross abnormalities affecting food safety and those affecting wholesomeness alone.

The acquisition of these data on a national industry scale is well beyond the capacity of any research team. While the core research team designed the assessments and monitored data quality, the collection of the data was dependent on a combination of engagement and access granted by industry, together with official inspectors working in abattoirs nationally.

The expert panels provided essential industry guidance on how national data prevalence and technique comparison data could be recorded by competent industry personnel on a standardised basis during routine operations. This fostered engagement and contributions by industry to meet sampling framework requirements (i.e., exposure assessment data) that included proportional regional slaughter numbers and captured regional disease and condition variation data for national assessments. The panels also assisted in estimating the *Se*, interpreting the data, and recommending validated amendments for formal risk management consideration.

The equivalence of alternative techniques was based on estimating adverse effects on food safety, wholesomeness, and the surveillance of animal health (including zoonoses) and welfare [3,14]. Quantitative metrics considered by the Competent Authority for the determination of equivalence of alternative post-mortem inspection techniques included comparisons of estimated public health risk, non-detection rates for gross abnormalities, and microbial contamination resulting from inspection activities, as appropriate.

The selection of methodologies to compare inspection procedures also followed a risk-based approach. For the classic meat-borne zoonosis *Cysticercus bovis,* a full quantitative risk assessment was conducted to predict the effect of different post-mortem inspection options on consumer risk. Whereas, for gross abnormalities affecting wholesomeness alone, non-detection rates were compared. Meat hygiene assessments were used to identify any beneficial microbiological effects on meat safety in assessments of sheep and pigs [27,29].

The publication of peer-reviewed papers from the review that accompanied the proposals (Figure 1) provided additional assurance to the Competent Authority when assessing equivalence for the amended standard. These publications also informed the notification and acceptance of equivalence to maintain export market access.

Alternative PMID procedures were accepted as equivalent with the standard by Competent Authority risk managers resulting in the amended AS4696:2023 [46], which was activated throughout domestic and export-licenced abattoirs from 1 July 2023. While assuring equivalent meat safety and wholesomeness, the amended standard facilitates reduced product wastage, the optimisation of carcase cuts, and serves as a basis for the allocation of official PMID resources commensurate with risk.

## Figures and Tables

**Table 1:** Disposition guidance for carcasses totally condemned with bovine respiratory disease complex [39].

Criteria	Acute Stage	Organising Stage	Resolving Stage	Chronic Stage
Disease stage	Bacteraemia/viraemia	Primary bacteraemia/viraemia, secondary pathogens localising to susceptible organs	Lesions localising	Lesions localised and then diminishing in extent until no residual tissue damage remains
Indicative period post-infection ^1^	0–14 days	7–14 days	10–28 days	>20 days
Ante-mortem	May be showing signs of fever, e.g., lethargy, reluctance to move, increased rate of breathing, elevated temperature.	May be showing signs of fever, e.g., lethargy, increased rate of breathing, elevated temperature. Possible muco-purulent nasal discharge. Cough, signs of discomfort on coughing	No fever, but possible muco-purulent nasal discharge. Possible cough.	Normal
Gross post-mortem abnormalities	Carcase showing signs of fever/septicaemia (e.g., erythema and/or petechial haemorrhages and/or poly-serositis with straw coloured exudate with fibrin clots in cavities).	Bronchopneumonia. Early fibrous adhesions between visceral (i.e., organ) and parietal (i.e., rib) serosal surfaces. Erythema of serosal surfaces. Possible purulent exudate.	Adhesions well developed. Serosal erythema mild to moderate. Exudates resolving, abscess formation. Possible arthritis.	Probable hyperaemia of serosa. Diffuse/localised pleurisy and peritonitis. Possible chronic abscess. Possible (poly) arthritis.
BRD agent present in primary lesion	Yes	Probably	Possibly	Unlikely
Septicaemic with primary BRD agent	Probably	Possibly	No	No
Muscle contaminated with primary BRD agent	Unlikely	No	No	No
Muscle contaminated with foodborne hazard	Possibly	Unlikely	No	No
Wholesomeness after stripping membranes	No	No	Possibly	Most likely
Disposition judgement	Total carcase condemnation	Total carcase condemnation	If uncertain—hold and test	Pass—trim affected parts

^1^ Indicative period for each stage, will vary considerably between animals, the effectiveness of treatments, and may vary in other countries pending aetiologies.

## Data Availability

The original contributions presented in the study are included in the article, further inquiries can be directed to the corresponding author.
